# Synthesis of RP 48497, an Impurity of Eszopiclone

**DOI:** 10.3390/molecules13081817

**Published:** 2008-08-22

**Authors:** Yu Sha, Lei Zhang, Gui-Jie Du, Jian Ren, Mao-Sheng Cheng

**Affiliations:** 1 Key Lab of New Drug Design and Discovery of Liaoning Province, School of Pharmaceutical Engineering, Shenyang Pharmaceutical University, Shenyang 110016, P. R. China; E-mails: shayu@syphu.edu.cn; zl.719@163.com; renjian_1981@hotmail.com; 2 Jinzhou Jiutai Pharmaceutical Co. Ltd, Jinzhou 121012, P.R. China; E-mail: duguijie@yahoo.com.cn

**Keywords:** RP 48497, eszopiclone, quality control

## Abstract

RP 48497 is a photodegradation product of eszopiclone, a non-benzodiazepine sedative-hypnotic used in the treatment of insomnia. We report herein the first synthesis of RP 48497 *via* reduction, chlorination, and recyclization of 6-(5-chloropyridin-2-yl)-7-hydroxy-6,7-dihydropyrrolo[3,4-b]pyrazin-5-one (**3**), a key intermediate in the synthesis of eszopiclone. The structure of RP 48497 was confirmed by its ^1^H-NMR and MS data. The mechanism of the reduction step in the synthesis of RP 48497 was also studied and the key parameters were determined. These findings should be important for quality control purposes in the manufacture of eszopiclone.

## Introduction

Eszopiclone [(+)-6-(5-chloro-2-pyridinyl)-(7*S*)-(4-methylpiperazin-1-yl-carbonyloxy)-6,7-dihydro-5H-pyrrolo[3,4-b]pyrazine-5-one, RP 27267, Estorra®, Lunesta®], the (*S*)-enantiomer of zopiclone, is more active and less toxic than the racemic zopiclone. It is metabolized by three major pathways, involving decarboxylation, oxidation and demethylation. The main urine metabolites are the less active eszopiclone-*N*-oxide (RP 29753) and the inactive *N*-desmethyleszopiclone (RP 32273). RP 29753 can also be considered as an impurity of eszopiclone and is formed by the oxidation of the latter. The related substance RP 29307 is formed during the penultimate phase of the synthesis. It may pre-exist in the starting material and it can also be formed by the hydrolysis of eszopiclone during storage. Finally, RP 48497 is a non-chiral potential photodegradation product whose formation is caused by bright light [1].

**Figure 1:**
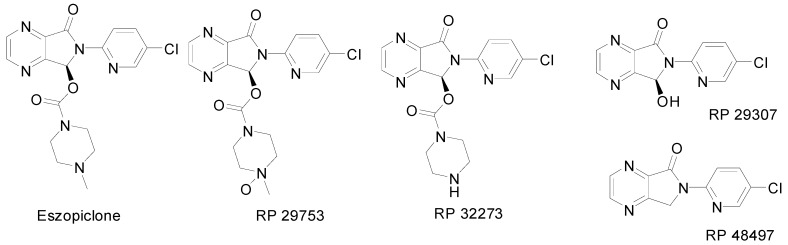
Structures of eszopiclone and its metabolites.

**Scheme 1:**
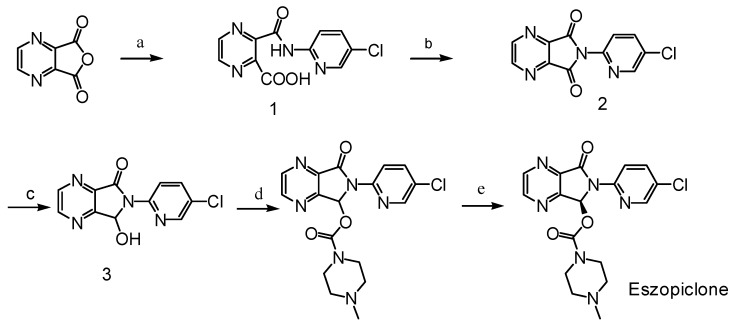
Synthetic route to eszopiclone.

As syntheses of the other impurities of eszopiclone, except RP 48497, have been previously reported, the main aim of our work presented here was to synthesize this compound. This is significant for the quality control of eszopiclone, whose chemical synthesis as shown in Scheme 1 has been reported [2,3,4,5]:

(a) 5-Chloropyridin-2-ylamine, CH_3_CN, reflux; (b) Ac_2_O, reflux; (c) KBH_4_, 1,4-dioxane, H_2_O, 13 ^◦^C; (d) 4-methylpiperazine-1-carbonyl chloride hydrochloride, Et_3_N, pyridine, CH_2_Cl_2_, reflux; (e) tartaric acid or malic acid

## Results and Discussion

### Synthesis

**Scheme 2:**
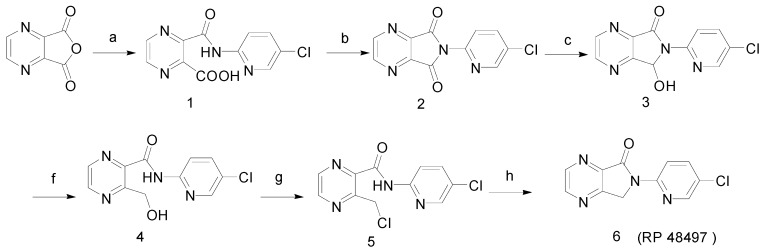
Synthetic route to RP 48497.

Our synthetic route to RP 48497 using the same starting materials as that to eszopiclone is illustrated in Scheme 2. Considering the instability of intermediate **3**, its treatment with excess KBH_4 _or NaBH_4_ resulted in the cleavage of the carbon-nitrogen bond to yield compound **4**. Finally, compound **5**, obtained by the chlorination of **4**, was intramolecularly cyclized without purification in the presence of NaH to form the target compound RP 48497 (**6**). The total yield was 40 %.

(a) 5-Chloropyridin-2-yl-amine, CH_3_CN, reflux; (b) Ac_2_O, reflux; (c) NaBH_4_, 1,4-dioxane, H_2_O, 13 ^◦^C; (d) 4-methylpiperazine-1-carbonyl chloride hydrochloride, Et_3_N, pyridine, CH_2_Cl_2_, reflux; (e) tartaric acid or malic acid; (f) NaBH_4_, 1,4-dioxane, H_2_O; (g) CH_2_Cl_2_, SOCl_2_; (h) NaH, DMF

**Scheme 3:**
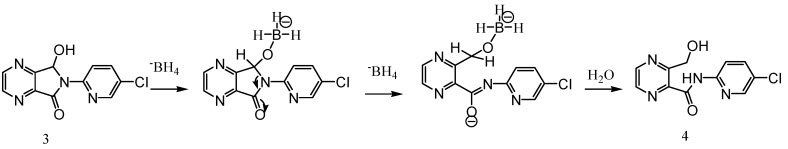
Mechanism of formation of intermediate **4**.

After studying in depth the reduction step, we found that the amount of reductant used played a vital role in the transformation of the intermediate **2** to the intermediate **3**. If more than 0.5 molecular equivalents of reductant were present in the reaction mixtures, a certain amount of a byproduct whose structure has been identified as compound **4** could be obtained, and the content of **4** would increase when either the reaction temperature or the ratio of the reductant were increased. The polarity of **3** and **4**, according to TLC, therefore, it is very difficult to separate them and purify of **3** from **4**. The mechanism of the transformation from **3** to **4** can be explained as shown in Scheme 3 [6]:

For this reason, the reduction of intermediate **2** to **3** is thus a key step in the preparation of eszopiclone. The content of compound **4** is a valid parameter for quality control of the intermediate **3**. Since this impurity **4** is mixed with the intermediate **3**, it could also be further acylated with 4-methylpiperazine-1-carbonyl chloride hydrochloride during the process to give compound **7** (Scheme cheme 4 ), a ring-opening derivative of eszopiclone.

**Scheme 4:**
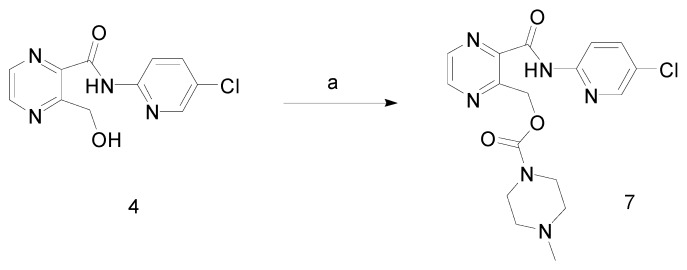
Formation of compound **7**.

(a) 4-Methylpiperazine-1-carbonyl chloride hydrochloride, Et_3_N, pyridine, CH_2_Cl_2_, reflux

Considering that it is very hard to remove compound **7** as a consequence of the similar polarities of eszopiclone and compound **7**, it may be concluded that careful control on the reduction step to prevent the formation of compound **4** is important in the quality control of eszopiclone.

## Experimental

### General

All commercially available reagents and solvents were used without further purification, unless specified. Solvents were dried and re-distilled prior to use according to standard methods. Melting points are uncorrected and were determined on a Büchi Melting Point B-540 apparatus. ^1^H-NMR spectra were recorded on a Bruker ARX 300 MHz instrument, using DMSO-*d*_6_ as solvent and TMS as the internal standard. Coupling constants (*J*) are expressed in Hz. Mass spectra were obtained on an Agilent 1100 mass spectrometer. Column chromatography (CC) was performed on silica gel H and analytical TLC on silica gel HF254.

### 3-Hydroxymethyl-pyrazine-2-carboxylic acid (5-chloro-pyridin-2-yl)-amide (**4**)

To a mixture of 1,4-dioxane (230 mL) and water (12 mL), was added 6-(5-chloropyridin-2-yl)-7-hydroxy-6,7-dihydropyrrolo[3,4-b]pyrazin-5-one (**3**, 26.4 g, 0.10 mol).. After cooling to 10 ^◦^C, NaBH_4 _(2.7 g, 0.07 mol) was added to the above suspension. The mixture was stirred for 2 hours with the temperature kept between 10 ^◦^C to 15 ^◦^C and then poured into ice/water (300 mL). Glacial acetic acid was added to adjust the pH to 5-6 and the mixture was stirred for 30 minutes. The precipitated solid was filtered and dried to give 20.0 g of a yellow product. Yield: 75.2 %; m.p.:176.0 ^◦^C-177.3 ^◦^C; ^1^H-NMR δ: 10.89 (1H, s), 8.84 (1H, d, *J* = 2.31), 8.69 (1H, d, *J* = 2.25), 8.45 (1H, d, *J* = 2.43), 8.26 (1H, d, *J* = 8.94), 8.03 (1H, dd, *J* = 2.61, 8.91), 5.34 (1H, t, *J* = 5.91), 4.93 (2H, d, *J* = 5.67).

### 3-Chloromethylpyrazine-2-carboxylic acid (5-chloro-pyridin-2-yl)-amide (**5**)

To a 0 ^◦^C suspension of 3-hydroxymethylpyrazine-2-carboxylic acid (5-chloropyridin-2-yl)-amide (**4**, 30.0 g, 0.10 mol) in dichloromethane (120 mL), thionyl chloride (42 mL) was added dropwise while keeping the temperature below 10 ^◦^C. After the addition, the mixture was stirred at room temperature for 2 hours and then evaporated to dryness under reduced pressure. The off-white product was recrystallized from *i*-propane to give brown needles, 21.0 g. Yield: 65.4 %; m.p.:149.0 ^◦^C-150.0 ^◦^C.

### 6-(5-Chloropyridin-2-yl)-6,7-dihydropyrrolo[3,4-b]pyrazin-5-one (RP 48497, **6**)

To a 0 ^◦^C suspension of NaH 4.3 g (70 %, 0.12 mol) in DMF (200 mL), 3-chloromethylpyrazine-2-carboxylic acid (5-chloropyridin-2-yl)-amide (**5**, 17.5 g, 0.06 mol) was added slowly. After the addition, the reaction mixture was stirred for 24 hours at room temperature. Additional NaH (70 %, 1.3 g, 0.04 mol) was then added and the reaction mixture was stirred for another 1 hour. The mixture was poured into ice/water (200 mL). After the ice dissolved, the precipitated solid was filtered to give 11.8 g of a yellow powder. Yield: 77.2 %. The crude product can be further purified by CC (petroleum ether-EtOAc, 10:1); m.p.: 222.0 ^◦^C-224.0 ^◦^C; ^1^H-NMR δ: 8.90 (2H, d, *J* = 2.60), 8.60 (1H, d, *J* = 9.00), 8.57 (1H, d, *J* = 2.58), 8.08 (1H, dd, *J* = 8.97, 2.64), 5.13 (2H, s); MS m/z: 47.2 [M+H]^+^, 269.1 [M+Na]^+^, 514.9 [2M+Na]^+^, 245.0 [M-H]^-^.
